# A Case of Malignant Pleural Effusion Secondary to Endometrial Cancer After One Year of Hysterectomy

**DOI:** 10.7759/cureus.28907

**Published:** 2022-09-07

**Authors:** Misbahuddin Khaja, Laura Yapor, Asim Haider, Muhammad Yasir Anwar, Diana M Ronderos, Dongmin Shin

**Affiliations:** 1 Internal Medicine/Pulmonary Critical Care, Icahn School of Medicine at Mount Sinai/BronxCare Health System, New York City, USA; 2 Internal Medicine, BronxCare Health System, New York City, USA

**Keywords:** malignancy, hysterectomy, metastasis, pleural effusion, endometrial carcinoma

## Abstract

Endometrial cancer is the most common malignant tumor of the female genital tract. It can rarely metastasize to the lung, presenting as a pulmonary nodule and pleural effusion. Here we present a case of a 76-year-old female with a history of endometrial cancer who underwent a total abdominal hysterectomy and came one year later for evaluation of shortness of breath. She was found to have pleural effusion. Diagnostic and therapeutic thoracentesis was positive for malignant cells originating from endometrial cancer. The patient could not tolerate chemotherapy due to poor functional status, and a tunnel pleural catheter was placed for symptomatic relief. In conclusion, it is a rare finding of malignant pleural effusion to have an origin as endometrial cancer. Pleura is the rare distant site of involvement from endometrial cancer.

## Introduction

Endometrial carcinoma is the most common gynecological neoplasm. It is estimated that 3.6% of patients with endometrial carcinoma develop lung metastases [1]. Out of all gynecologic malignancies, endometrial carcinoma has the highest frequency of pulmonary metastases. Metastatic endometrial carcinoma to the lungs most commonly presents with pulmonary nodules [2]. A malignant effusion is less commonly found in metastatic endometrial carcinoma of the lungs [1,2]. Uterine cancer most frequently spreads to para-aortic and pelvic lymph nodes. The spread to the pleura or lung is hematogenous. The other less common sites of metastasis from endometrial cancer are paranasal sinuses, heart, brain, and scalp [3].

## Case presentation

**Figure 1:**
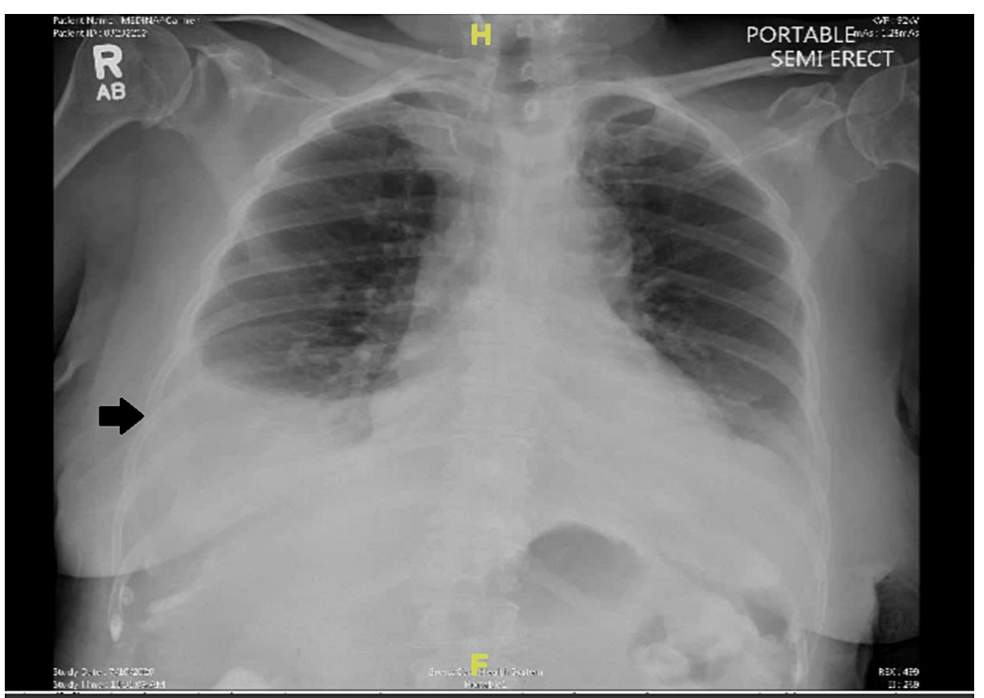
Chest radiograph showing right pleural effusion (black arrow).

**Figure 2:**
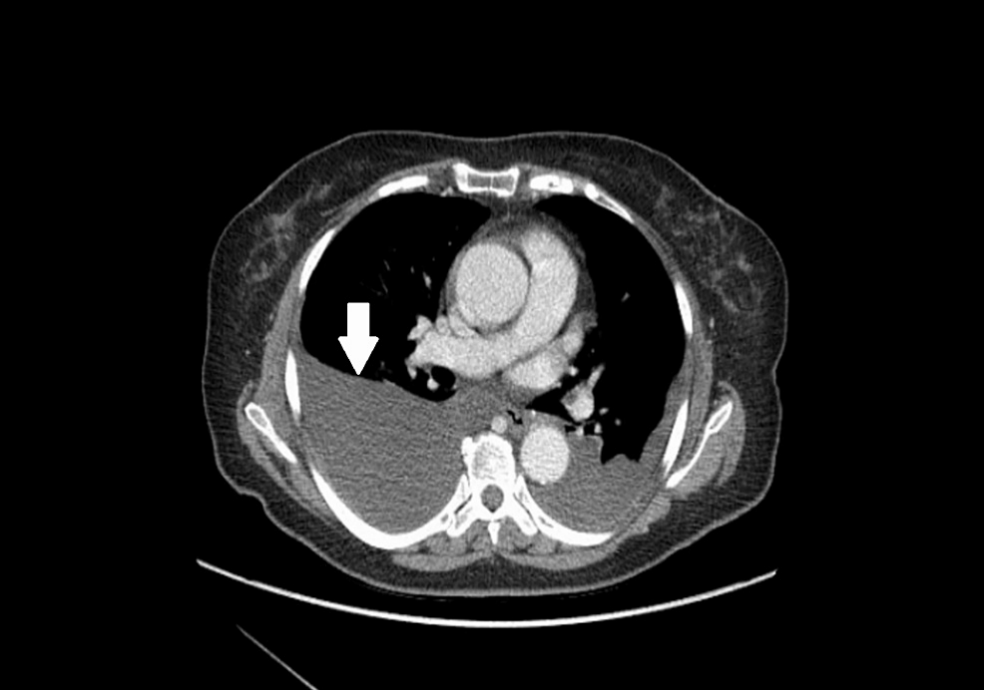
CT scan of the chest: axial view showing right pleural effusion greater (white arrow) than left.

A 76-year-old female patient presented to the hospital for evaluation of shortness of breath and was found to have bilateral pleural effusions seen in a CT scan of the chest. Her comorbidities included asthma, hyperlipidemia, neuropathy, and stage IA endometrial papillary serous adenocarcinoma (treated by total hysterectomy with bilateral salpingo-oophorectomy one year before this presentation). On presentation, her vital signs included a blood pressure of 130/89 mmHg, pulse rate of 92 beats per minute, respiratory rate of 22 per minute, a temperature of 98 degrees Fahrenheit, and oxygen saturation of 92% on room air. She was in no acute distress and was able to speak in complete sentences. However, she had decreased breath sounds in the bilateral lower lung field on physical examination. On presentation, chest X-ray (Figure 1) showed bilateral pleural effusion, and CT chest (Figure 2) revealed moderate to large right and moderate left pleural effusion.

**Figure 3:**
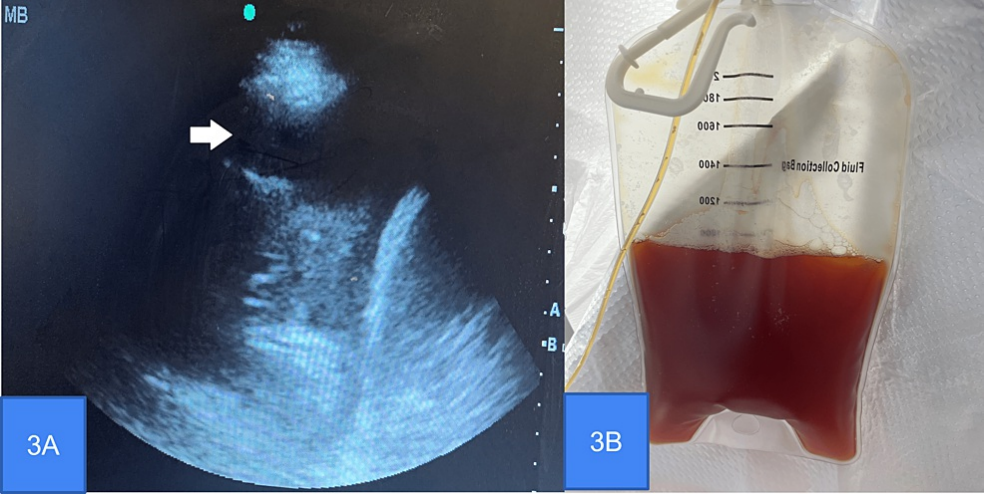
(A) Ultrasound image showing pleural effusion (white arrow); (B) Pleural drainage post thoracentesis showing serosanguineous pleural effusion.

A bedside ultrasound (Figure 3A) was performed; a large pleural effusion was seen. Her complete blood count, basic metabolic panel, liver function tests, and coagulation profile were within normal limits. Thoracentesis under sonographic guidance was performed, and 900 ml of serosanguinous fluid was drained (Figure 3B).

**Figure 4:**
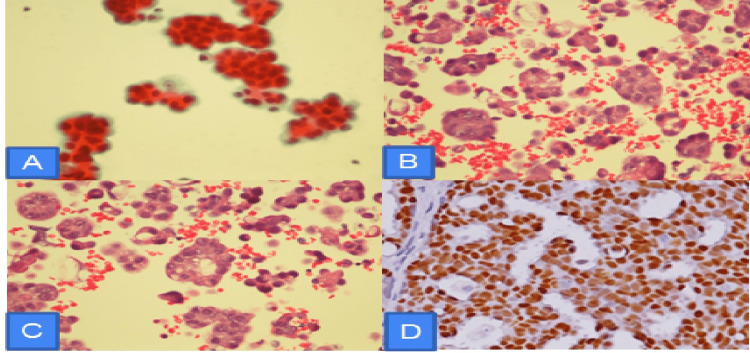
(A) Pleural fluid cell block showing cluster of single and group of atypical malignant cells with papillary configuration, consistent with endometrial adenocarcinoma as primary; (B, C) H&E x400 malignant cells with papillary serous endometrial carcinoma; (D) Immunohistochemical stain positive for estrogen receptors.

The aerobic and anaerobic pleural fluid cultures, as well as mycobacterial pleural fluid cultures, were negative. The cytology of the pleural fluid reported single groups of malignant cells with papillary configurations, consistent with the endometrial primary. Following the cytology report, cell block pathology was done, reporting malignant cells consistent with serous carcinoma. Immunohistochemical stains were positive for estrogen and negative for progesterone (Figure 4).

The patient was later discharged home with a permanent indwelling pleural catheter with an oncology follow-up appointment.

## Discussion

The pleura produces approximately 0.26 ml/kg/day of body weight of pleural fluid [4]. An imbalance of production and absorption can occur in metastatic disease with carcinosis, which leads to increased production of abnormal pleural fluid [1,2]. Endometrial carcinoma is the most common malignant tumor of the female genital tract and the fourth most common tumor in women, with dissemination occurring in 2-4% of cases [4].

In serous papillary endometrial carcinoma, distal metastases usually spread hematogenously. Uterine malignancies most commonly spread to pelvic and paraaortic nodes, whereas distant spread to thoracic metastasis usually spreads via a hematogenous system [5,6]. Additionally, pleural effusion has a higher association with squamous cell carcinoma and a lower association with adenocarcinoma. It is rare for endometrial cancer to present with pleural effusions, and few studies have discussed the incidence of pleural metastasis from gynecologic malignancies [7].

The parietal pleura is more involved in the pleural fluid exchange than the visceral pleura, most likely due to its closeness to microvessels and lymphatics, where malignancies can spread [8]. In addition, metastatic malignant pleural effusions are exacerbated by carcinosis, which creates direct irritation and subsequently increases pleural fluid production [9].

In cases of metastatic disease, malignant cells of the visceral and parietal pleura incite the production of abnormal pleural fluid, with lung metastases common in the later stages of malignant disease and producing a poor prognosis. A total of 15% of malignant neoplasms lead to pleural effusions [2]. The cytopathologic diagnosis of pleural fluid by Johnston WW reported that female genital tract-induced malignant pleural effusions, such as adenocarcinoma of endometrium and ovary, are the most common. Endometrium accounted for eight of the 38 metastatic tumors of the female genital tract, and the ovary was 29 [10].

The characteristics of metastatic pleural effusions include exudative, bloody, and lymphocytic effusions, with additional pleural fluid cytology, which usually confirms malignant cells [7]. To determine if endometrial cancer has metastasized to the pleura or lung, it must be differentiated from mesothelioma via immunohistochemistry [1]. Branscheid D et al. found that 2.2% of pleural effusions stemmed from uterine/ovarian cancers in 414 patients [11].

Treatment involves surgical cytoreduction followed by systemic therapy for patients with metastatic endometrial cancer [12]. Patients who are surgical candidates and undergo cytoreduction should get adjuvant chemotherapy with carboplatin and paclitaxel. Nonsurgical candidates should get medical therapy. Those patients have a five-year survival of less than 20% [12]. In a study by Albright BB et al., which included 3600 patients with advanced endometrial cancer who underwent cytoreduction surgery, complete cytoreduction with no residual disease was seen in 52% of patients, while residual disease of less than one centimeter was seen in 75% [13].

Patients with estrogen receptor-positive cancer who have progressed on chemotherapy or immunotherapy and want to avoid chemotherapy toxicities may use endocrine therapy as alternative first or second-line therapy [14]. The options for treatment of malignant pleural effusion include simple drainage, tunnel pleural catheter, pleurodesis, and pleurectomy [15].

## Conclusions

Pleura is a rare site of metastasis for endometrial carcinoma. Therefore, physicians should consider the possibility of endometrial carcinoma in females presenting with pleural effusion. Cytopathological examination of the pleural fluid for the presence of malignant cells is an essential diagnostic tool in this regard. In addition, moderate-to-severe pleural effusions in patients with gynecological malignancies should raise suspicion of metastatic pleural disease.
